# Diagnostic usefulness of an amino acid tracer, α-[*N*-methyl-^11^C]-methylaminoisobutyric acid (^11^C-MeAIB), in the PET diagnosis of chest malignancies

**DOI:** 10.1007/s12149-013-0750-4

**Published:** 2013-07-04

**Authors:** Ryuichi Nishii, Tatsuya Higashi, Shinya Kagawa, Yoshihiko Kishibe, Masaaki Takahashi, Hiroshi Yamauchi, Hideki Motoyama, Kenzo Kawakami, Takashi Nakaoku, Jun Nohara, Misato Okamura, Toshiki Watanabe, Koichi Nakatani, Shigeki Nagamachi, Shozo Tamura, Keiichi Kawai, Masato Kobayashi

**Affiliations:** 1 Shiga Medical Center Research Institute, 5-4-30 Moriyama, Moriyama, Shiga 524-8524 Japan; 2 Department of Thoracic Surgery, Shiga Medical Center, 5-4-30 Moriyama, Moriyama, Shiga 524-8524 Japan; 3 Department of Respiratory Medicine, Shiga Medical Center, 5-4-30 Moriyama, Moriyama, Shiga 524-8524 Japan; 4 Department of Radiology, Faculty of Medicine, Miyazaki University, 5200 Kihara, Kiyotake-cho, Miyazaki, Miyazaki 889-1692 Japan; 5 Division of Health Sciences, Graduate School of Medical Science, Kanazawa University, 5-10-80 Kodachino, Kanazawa, Ishikawa 920-0942 Japan

**Keywords:** Amino acid tracer, Methylaminoisobutyric acid, Fluorodeoxyglucose, Lung cancer, Lymphadenopathy, Positron emission tomography, Sarcoidosis

## Abstract

**Objectives:**

Although positron emission tomography (PET) using [^18^F]-fluoro-2-deoxy-d-glucose (^18^F-FDG) is established as one of the first-choice imaging modalities in the diagnosis of chest malignancies, there are several problems to solve in clinical practice, such as false positive uptake in inflammatory diseases. The aim of this study was to evaluate the clinical usefulness of an amino acid tracer, α-[*N*-methyl-^11^C]-methylaminoisobutyric acid (^11^C**-**MeAIB), in the diagnosis of chest malignancies, in combination with ^18^F-FDG.

**Setting:**

Fifty-nine cases (57 patients, 66 ± 12 years old) who consulted to our institution for the wish to receive differential diagnosis of chest diseases were included. Purpose of the studies were as follows: differential diagnosis of newly developed lung nodules, *n* = 22; newly developed mediastinal lesions, *n* = 20; and both, *n* = 17 (including lung cancer: *n* = 19, lymphoma: *n* = 1, other cancers: *n* = 2, sarcoidosis: *n* = 15, non-specific inflammation: *n* = 18, other inflammatory: *n* = 4, respectively). Whole-body static PET or PET/CT scan was performed 20 and 50 min after the IV injection of ^11^C-MeAIB and ^18^F-FDG, respectively.

**Results:**

^11^C-MeAIB uptake of malignant and benign lesions was statistically different both in pulmonary nodules (*p* < 0.005) and in mediastinal lesions (*p* < 0.0005). In visual differential diagnosis, ^11^C-MeAIB showed higher results (specificity: 73 %, accuracy: 81 %), compared to those in ^18^F-FDG (60, 73 %, respectively). In cases of sarcoidosis, ^11^C-MeAIB showed higher specificity (80 %) with lower uptake (1.8 ± 0.7) in contrast to the lower specificity (60 %) with higher uptake of ^18^F-FDG (7.3 ± 4.5).

**Conclusions:**

^11^C-MeAIB PET/CT was useful in the differential diagnosis of pulmonary and mediastinal mass lesions found on CT. ^11^C-MeAIB PET or PET/CT showed higher specificity than that of ^18^F-FDG PET/CT in differentiating between benign and malignant disease. Our data suggest that the combination of ^18^F-FDG and ^11^C-MeAIB may improve the evaluation of chest lesions, when CT and ^18^F-FDG PET/CT are equivocal.

## Introduction

The positron emission tomography (PET) radiopharmaceutical 2-deoxy-2-[^18^F]fluoro-d-glucose (^18^F-FDG) is currently used for diagnostic imaging of a variety of tumors [1]. However, ^18^F-FDG is known to accumulate in normal structures and in sites of inflammation, reducing its specificity [2–5]. Several investigators have reported that ^18^F-FDG PET performed to assess for malignancy showed 20–50 % false positive results, with the higher false positive rates found in geographical areas of endemic granulomatous diseases, such as histoplasmosis or tuberculosis [5–8].

A variety of new tracers has been introduced in order to overcome these limitations [9–11]. Amino acid tracers are one promising category. ^11^C-methionine (^11^C-MET) has been extensively investigated, though with several drawbacks being revealed [12]. ^11^C-MET is not stable in vivo, becoming *trans*-methylated, and losing its ^11^C moiety as it is metabolized [13]. Several studies have shown that ^11^C-MET is not tumor-specific, and accumulates in some inflammatory diseases, such as sarcoidosis [14, 15].

[*N*-methyl-^11^C]α-methylaminoisobutyric acid (^11^C-MeAIB) is an artificial amino acid PET tracer, which, unlike ^11^C-MET, is metabolically stable in vivo [16, 17]. It has been shown that ^11^C-MeAIB is a useful tracer for measurement of amino acid uptake by skeletal muscle, and in the diagnosis of malignant lymphoma and head and neck cancers [16, 18, 19]. The utility of ^11^C-MeAIB PET in the diagnosis of chest malignancies has not yet been evaluated.

The purpose of this study is to investigate the efficacy of ^11^C-MeAIB PET or PET/CT as a diagnostic tool for distinguishing between malignancies and inflammatory diseases, when CT or ^18^F-FDG PET or PET/CT shows equivocal findings.

## Materials and methods

### Patients

From March 2009 to September 2012, 59 cases (57 patients; male: 35, female: 22; ranging from 28 to 88 years; mean age: 65.5 ± 12.3) (Table 1, left) of new chest lesions detected by CT scan (21 patients had lung lesions only, 20 patients had mediastinal lesions only, and 18 patients had both lesions) were selected. Two patients received this study protocol twice with 1 year interval or more. Each patient gave written informed consent. The tracer study was approved by our institutional review boards, the Human Study Committee (approval number: #36-04, Mar. 25, 2009) and the Committee for the Clinical Use of Short-Half Life Radioactive Materials (approval number: #2008-01, Nov. 28, 2008).

**Table 1:** Characteristics of Total 57 Patients [59 PET studies (2 patients received 2 series of PET studies with >5 months interval)] and 80 lesions

Age (years)		Final diagnosis (lesion-based), *n* (%)	
Mean ± SD	65.5 ± 12.3		
Range	28–88	Pulmonary nodules in the lung fields: (*n* = 42)	
Gender, *n* (%)		Lung cancer	18 (43 %)
Male	35 (61 %)	Adenocarcinoma	12 (29 %)
Female	22 (39 %)	Squamous cell carcinoma	3 (7 %)
		Small cell carcinoma	2 (5 %)
Purpose of total 57 PET study, *n* (%)		Large cell carcinoma	1 (2 %)
Evaluation of lung nodule(s)	22 (37 %)	Other malignancies (post-treated)	4 (10 %)
Evaluation of mediastinal nodule(s)	20 (34 %)	Mesothelioma	2 (5 %)
Evaluation of both	17 (29 %)	Colorectal cancer	1 (2 %)
		Lymphoma	1 (2 %)
Baseline diseases, *n* (%)		Non-malignant diseases	20 (48 %)
None, newly developed lung nodule	30 (51 %)	Non-specific inflammatory changes	13 (31 %)
None, newly developed mediastinal lesion(s)	20 (34 %)	Sarcoid nodule	5 (12 %)
Newly developed chest lesion(s)		Abscess	2 (5 %)
in the follow-up of other malignancies (post operated)	9 (15 %)		
Gastric malignant lymphoma	2 (3 %)	Hilar and mediastinal nodules: (*n* = 38)	
Colorectal cancer	3 (5 %)	Malignant diseases	10 (26 %)
Gastric cancer	2 (3 %)	Adenocarcinoma	4 (11 %)
Esophageal cancer	1 (2 %)	Squamous cell carcinoma	3 (8 %)
Urinary bladder cancer	1 (2 %)	Small cell carcinoma	1 (3 %)
		Mesothelioma	1 (3 %)
Final confirmation of 57 PET diagnosis		Lymphoma	1 (3 %)
Pathological confirmation	35 (59 %)	Non-malignant diseases	28 (74 %)
Surgical resection (including VATS**)	17 (29 %)	Sarcoidosis	15 (39 %)
Biopsy at bronchoscopy	6 (10 %)	Non-specific inflammatory changes	9 (24 %)
Biopsy at thoracoscopy	3 (5 %)	IgG4 related disease	2 (5 %)
CT-guided biopsy	1 (2 %)	Squamous cell papilloma	1 (3 %)
Resection or biopsy of superficial lymph node	7 (12 %)	Tubercular nodule	1 (3 %)
Sputum culture	1 (2 %)		
Clinical follow-up (>12 months)	24 (42/1 %)		

Inclusion criteria were: (1) a new chest lesion detected on CT scan, suspicious for malignancy, (2) CT (and ^18^F-FDG PET or PET/CT) showing equivocal findings, with further evaluation requested by referring pulmonologists, and (3) disease confirmed by pathology, or clinical follow-up for more than 12 months after PET studies. Exclusion criteria were: (1) malignant or inflammatory lesions which received treatment within 6 months before ^18^F-FDG PET or PET/CT, (2) apparent direct invasion of neighboring organs, (3) apparent extra-pulmonary metastatic lesions, and (4) patients who refused to undergo ^11^C-MeAIB PET or PET/CT. Baseline diseases or detailed background of clinical conditions are shown in Table 1 (left).

The CT scans had been performed as routine clinical studies with a multi-detector row CT scanner, Aquilion 16 (Toshiba, Tokyo, Japan). All patients subsequently underwent both ^18^F-FDG and ^11^C-MeAIB PET or PET/CT within 2 weeks of the CT.

### PET studies

^18^F was produced by ^18^O(p, n) ^18^F reaction. ^18^F-FDG was synthesized by the nucleophilic substitution method using an ^18^F-FDG-synthesizing instrument F-100 (SHI, Tokyo, Japan) and a cyclotron, CYPRIS-325R (SHI, Tokyo, Japan). Otherwise, ^18^F-FDG was purchased commercially from Nihon-Medi-Physics (Tokyo, Japan).

Production of ^11^C-MeAIB was based on the method proposed by Någren et al. [20]. The patients fasted for more than 5 h before the injection of ^18^F-FDG or ^11^C-MeAIB. Blood glucose levels were measured before the injection of ^18^F-FDG, and all the patients showed levels of ≤150 mg/dL (95.2 ± 13.1 mg/dL). All subjects underwent two separate scans, one following an intravenous injection of ^18^FDG (298 ± 68 MBq), and another after the intravenous injection of ^11^C-MeAIB (512 ± 50 MBq). Whole-body PET image acquisition commenced 50 min after ^18^F-FDG and 20 min after ^11^C-MeAIB injection. PET scans were performed either by a whole-body PET scanner, GE Advance (GE Healthcare, Waukesha WI, USA), or by a whole-body PET/CT scanner, Siemens True Point Biograph 16 (Siemens/CTI, Erlangen, Germany).

### Image analysis

Visual analysis of each lesion on the two PET scans was performed by two experienced nuclear medicine physicians (RN, TH) provided with clinical information including CT scans and tumor markers. All lesions were graded as positive or negative by consensus of two readers. If a nodule showed similar or lower uptake than that in the upper to middle normal mediastinal tissues, uptake was defined as negative. If a nodule showed higher uptake than that of the normal mediastinal tissues, its uptake was defined as positive. However, high ^18^F-FDG uptake was sometimes defined as negative in cases of sarcoidosis and other benign entities with a characteristic pattern by consensus of two readers.

Semi-quantitative analysis of ^18^F-FDG and ^11^C-MeAIB uptake was also performed. Regions of interest (ROIs) were defined on the target lesions in the transaxial tomograms of PET-only images. PET-to-CT co-registration was performed using automatic rigid/non-rigid body-deformable fusion software: Quantiva/BodyGuide (Tomographix IP Ltd., Toronto, Canada). In PET/CT scans, ROIs were defined and confirmed on the fused PET and CT images (hereafter all scans will be referred to as PET/CT scans, since PET images were always fused to CT images). The standardized uptake value (SUV) was calculated as follows:

$$ {\text{SUV}}\,{ = }\,\frac{{{\text{C}}\left( {{\text{k}}{{\text{Bq}} \mathord{\left/ {\vphantom {{\text{Bq}} {\text{ml}}}} \right. \kern-0pt} {\text{ml}}}} \right)}}{{{{{\text{ID}}\left( {\text{kBq}} \right)} \mathord{\left/ {\vphantom {{{\text{ID}}\left( {\text{kBq}} \right)} {{\text{body}}\,{\text{weight}}\,\left( {\text{g}} \right)}}} \right. \kern-0pt} {{\text{body}}\,{\text{weight}}\,\left( {\text{g}} \right)}}}} $$

where C represents tissue activity concentration measured by PET and ID represents the injected dose. The mean SUV of the normal tissue (lung field and mediastinum) was defined as the SUVmean. The highest SUV of the lesion was defined as the SUVmax.

### Statistics

All values are expressed as mean ± SD. All the statistical analyses were performed using statistical software, JMP 8J version (SAS Institute, Cary NC, USA), in which *p* values <0.05 were considered statistically significant. A comparison between each group was analyzed by the Wilcoxon score for unpaired data. Receiver operating characteristic curve (ROC) analyses for the diagnostic accuracy in ^11^C-MeAIB PET/CT and ^18^F-FDG PET/CT were generated using GraphPad Prism ver. 5.0 (GraphPad software, San Diego CA, USA). A comparison of SUVmax between ^11^C-MeAIB and ^18^F-FDG in each lesion was analyzed by the Logistic regression.

## Results

Table 1 summarizes the patient characteristics. The final diagnosis was confirmed pathologically by surgical resection in 17 cases, while biopsy at bronchoscopy, thoracoscopy, and CT-guided biopsy confirmed diagnosis in 10 cases. Resection or biopsy of lymph nodes at neck or supra-clavicular area was performed in seven cases. Clinical diagnosis was determined by follow-up for at least 12 months in 24 cases. Of the 59 cases in the present study, there were 22 malignant and 20 benign pulmonary nodules in the lungs and 10 malignant and 28 benign mediastinal lesions.

**Fig. 1:**
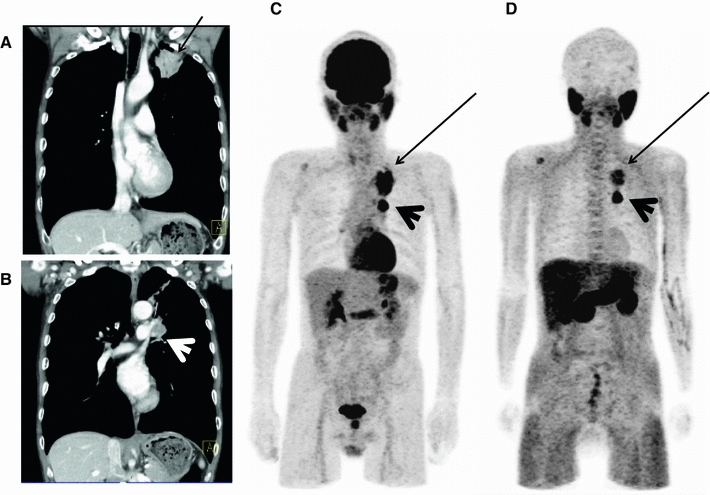
A case of lung cancer. Fifty-nine-year-old asymptomatic male patient. Screening chest X-ray showed abnormal density in the left upper lung zone. The patient underwent a contrast-enhanced chest CT scan, which revealed a nodule in the left apex (**a**) with probably lymph node (ln) metastasis in the *left* hilum (**b**). Bronchoscopic biopsy revealed squamous cell carcinoma (SCC). Both ^18^F-FDG PET and ^11^C-MeAIB PET (**c**, **d**: MIP image) showed high accumulation in the left upper lobe lesion (SUVmax = 13.2 for ^18^FDG, 6.3 for ^11^C-MeAIB, *arrows*) and *left* hilar ln (SUVmax = 21.9 for ^18^F-FDG, 11.5 for ^11^C-MeAIB, *arrowheads*). It should be noted that ^18^F-FDG uptake was relatively greater in the periphery than that of ^11^C-MeAIB. It was confirmed as concomitant inflammatory changes in the tissue surrounding the lesion. A small bone metastasis was also suspected in the right scapula (not pathologically confirmed)

**Fig. 2:**
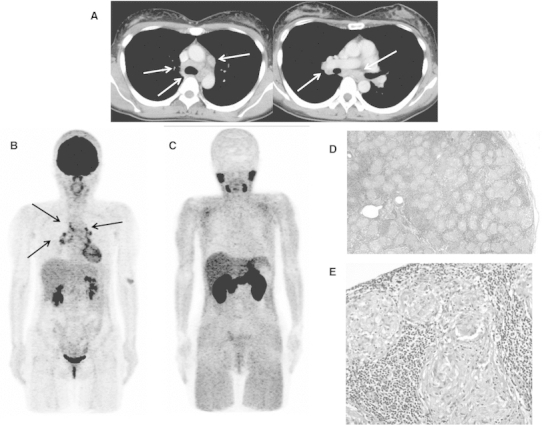
A case of sarcoidosis. Forty-four-year-old asymptomatic female patient. She had been followed as an outpatient after complete remission (more than 1 year previously) of malignant abdominal lymphoma (post-chemotherapy). She had no history of sarcoidosis or uveitis. The patient underwent chest CT scan (**a**), which revealed multiple nodules (*arrows*) in the middle mediastinum. ^18^F-FDG PET (**b**: MIP image) showed high accumulation in these lesions (SUVmax = 6.0 for the highest uptake in right hilar lesion), but there was no other lesions in the body. ^11^C-MeAIB PET (**c**: MIP image) showed no accumulation in these lesions (SUVmax = 1.8, with the highest uptake in the mediastinum). Thoracoscopic biopsy revealed sarcoidosis (**d**: low magnification, **e**: high magnification)

**Fig. 3:**
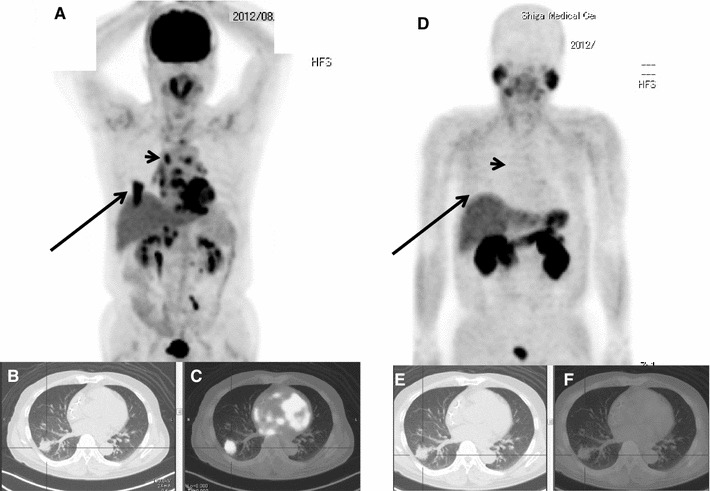
A case of sarcoidosis with lung nodule and mediastinal lesions. Sixty-three-year-old asymptomatic male patient. He had been followed as an outpatient for mediastinal sarcoidosis in another hospital. A new lung nodule was detected on CT scan and he was referred to our hospital. Both ^18^F-FDG PET/CT and ^11^C-MeAIB PET/CT were performed (**a**, **d**: MIP image). There was high ^18^F-FDG accumulation (SUVmax = 12.3) in the right lower lobe lesion (**b**: CT, **c**: PET/CT image), multiple mediastinal lesions, including right hilar lymph nodes (SUVmax = 7.6, *arrowheads*) and also in the upper abdominal lymph nodes (cardiac involvement was also indicated and confirmed later). ^11^C-MeAIB PET/CT showed very low accumulation (SUVmax = 1.7) in the right lower lobe lesion (**e**: CT, **f**: PET/CT image) (SUVmax = 1.7), multiple mediastinal lesions, including left hilar lymph nodes (SUVmax = 1.7, *arrowheads*), and also in the upper abdominal lymph nodes. Follow-up CT scan revealed shrinkage of lung nodule, and the diagnosis of sarcoidosis was confirmed

**Fig. 4:**
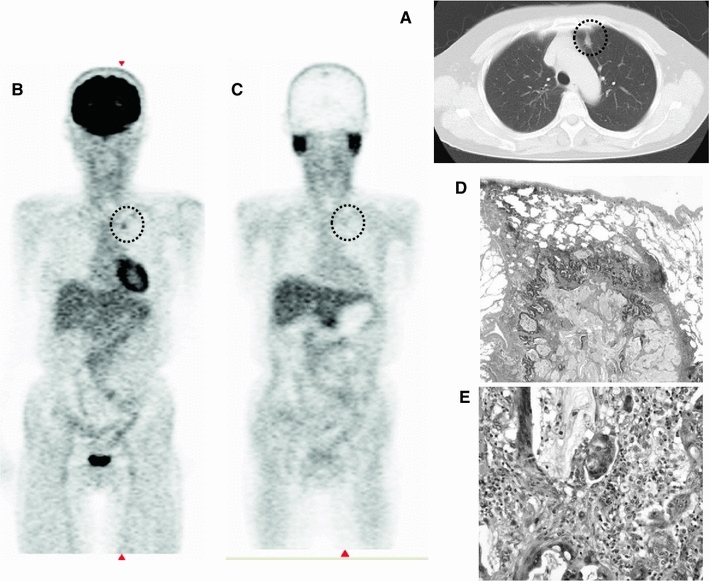
A case of metastatic colon cancer. Sixty-two year-old asymptomatic female patient and without elevated tumor markers. She had been followed as an outpatient after the resection of primary advanced colon adenocarcinoma with mucin secretion. In the follow-up period (1 year after surgery), the patient underwent a contrast-enhanced chest CT scan (**a**), which revealed a small lung nodule in the left apex. Both ^18^FDG and ^11^C-MeAIB PET showed faint accumulation in the left upper lobe lesion (SUVmax = 3.12 for ^18^F-FDG, 2.06 for ^11^C-MeAIB). Both ^18^F-FDG and ^11^C-MeAIB PET diagnoses were “equivocal” (visual diagnosis: negative, quantitative diagnosis: positive) (**b**, **c**). After close follow-up by CT scan and tumor markers, she underwent left upper lobe resection, and this lesion was confirmed as metastatic colon cancer with mucin secretion by pathology (**d**: low magnification, **e**: high magnification)

Figure 1 shows a typical malignant case, while Fig. 2 shows a typical benign case; in this instance, sarcoidosis. In Fig. 3, a ^18^F-FDG-strongly positive lung nodule was diagnosed as sarcoidosis. Equivocal findings of metastatic colon cancer are shown in Fig. 4.

### Semi-quantitative analysis of ^18^FDG uptake and ^11^C-MeAIB uptake

The average SUVmax and SUVmean for ^18^F-FDG and ^11^C-MeAIB uptake in normal lung and mediastinum are shown in Table 2. The average SUVmax of ^18^F-FDG in malignant lesions was significantly higher than that in benign lesions for 42 pulmonary nodules, while not significantly different for 38 benign and malignant mediastinal lesions. There was a wide overlap in ^18^F-FDG uptake between malignant and benign chest diseases, resulting in many false positive cases on ^18^F-FDG PET/CT, especially in mediastinal lesions.

**Table 2:**
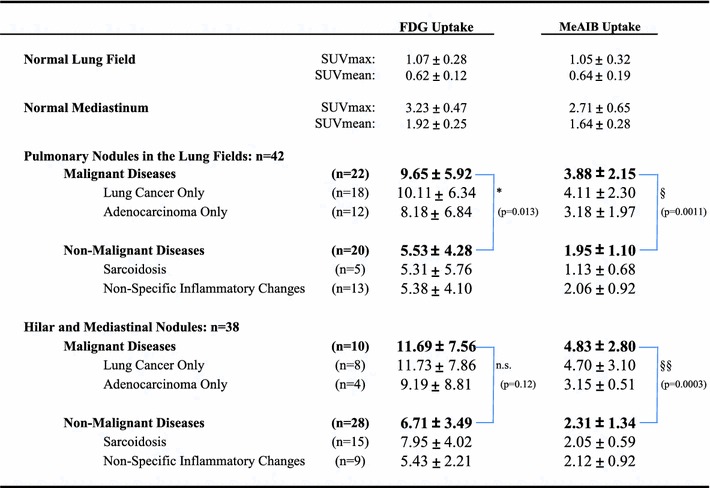
Quantitative analysis of total 42 pulmonary lesions and 38 mediastinal lesions

*n.s.* not significant

* *p* < 0.05, ^§^ *p* < 0.005, ^§§^ *p* < 0.0005

The average SUVmax of malignant lesions with ^11^C-MeAIB PET/CT was significantly lower than that for ^18^F-FDG PET/CT. On the other hand, ^11^C-MeAIB uptake by malignant and benign lesions showed greater statistical differences both among pulmonary nodules and mediastinal lesions.

**Fig. 5:**
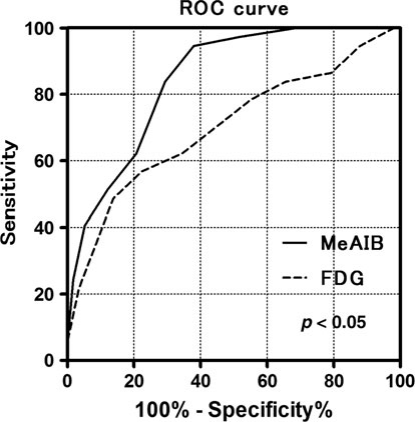
Receiver operating characteristic curve (ROC) analyses for the diagnostic accuracy of ^11^C-MeAIB and ^18^F-FDG using semi-quantitative analysis. The area under the curve (AUC) value for ^11^C-MeAIB PET/CT was 0.85 with standard error: 0.039, 95 % CI: 0.77–0.92 and *p* < 0.0001. AUC for ^18^F-FDG PET/CT was 0.70 with standard error: 0.056, 95 % CI: 0.60–0.82 and *p* < 0.001. These analyses indicated the better diagnostic accuracy of ^11^C-MeAIB for chest diseases (*p* < 0.05)

Figure 5 shows the result of ROC analyses for ^18^F-FDG and ^11^C-MeAIB, in which the diagnostic accuracies were obtained from the SUVmax values of each tumor in both PET/CT studies. An SUVmax = 3.0 was used as the threshold for ^18^F-FDG diagnosis. Other thresholds (SUVmax = 2.0, 2.5, 3.5, 4.0) gave similar or worse diagnostic results. In ^11^C-MeAIB PET/CT studies, an SUVmax = 2.0 was used as the optimum threshold. ^11^C-MeAIB scans showed a higher value than ^18^F-FDG scans both in patient-based (Fig. 5) and lesion-based diagnoses (not shown).

### Patient-based diagnostic results

Visual diagnosis of ^18^FDG and ^11^C-MeAIB on a per-patient basis in 59 cases is shown in Table 3 (top). The accuracy of ^18^FDG was 72.9 %, which was better than that from semi-quantitative analysis. Although ^18^FDG uptake was often equivocally positive, the final diagnosis was judged as true negative in many benign or inflammatory cases because of the analysis of pattern and location of the ^18^F-FDG uptake. Fifteen false positive cases with ^18^F-FDG were as follows: granulomatous inflammatory lung nodules and mediastinal lymphadenopathy confirmed by surgical resection (*n* = 6), mediastinal sarcoid lymphadenopathy by lymph node biopsy (*n* = 5), mediastinal IgG4-related lymphadenopathy by lymph node biopsy (*n* = 1), and non-specific inflammatory change followed for more than 12 months (*n* = 3). The accuracy of ^11^C-MeAIB was 81.4 %. Ten false positive cases with ^11^C-MeAIB were as follows: granulomatous inflammatory lung nodules and mediastinal lymphadenopathy confirmed by surgical resection (*n* = 4), mediastinal sarcoid lymphadenopathy by lymph node biopsy (*n* = 3), mediastinal IgG4-related lymphadenopathy by lymph node biopsy (*n* = 1), and non-specific inflammatory change followed for more than 12 months (*n* = 2). Nine of these ^11^C-MeAIB false positive cases were also false positive with ^18^F-FDG. One false negative case was of metastatic colon cancer, which was also false negative in ^18^F-FDG PET images (Fig. 4).

**Table 3:** Patient-based diagnostic results of FDG- and MeAIB-PET (PET/CT)

Visual diagnosis									
FDG (visual diagnosis)					MeAIB (visual diagnosis)				
	Patients with malignant lesions, *n* = 22	Patients with benign lesions, *n* = 37				Patients with malignant lesions, *n* = 22	Patients with benign lesions, *n* = 37		
FDG: positive	21	15	58.3	PPV (%)	MeAIB: positive	21	10	67.7	PPV (%)
FDG: negative	1	22	95.7	NPV (%)	MeAIB: negative	1	27	96.4	NPV (%)
	95.5	59.5	72.9			95.5	73.0	81.4	
	Sensitivity (%)	Specificity (%)	Accuracy (%)			Sensitivity (%)	Specificity (%)	Accuracy (%)	

Diagnosis using semi-quantitative analysis									
FDG (SUVmax = 3.0 as threshold)					MeAIB (SUVmax = 2.0 as threshold)				
	Patients with malignant lesions, *n *= 22	Patients with benign lesions, *n* = 37				Patients with malignant lesions, *n* = 22	Patients with benign lesions, *n* = 37		
FDG: positive	20	29	40.8	PPV (%)	MeAIB: positive	20	13	60.6	PPV (%)
FDG: negative	2	8	80.0	NPV (%)	MeAIB: negative	2	24	92.3	NPV (%)
	90.9	21.6	47.5			90.9	64.9	74.6	
	Sensitivity (%)	Specificity (%)	Accuracy (%)			Sensitivity (%)	Specificity (%)	Accuracy (%)	

*PPV* positive predictive value, *NPV* negative predictive value

In the semi-quantitative diagnoses (59 cases) with SUVmax = 3.0 cut-off value, the accuracy of ^18^F-FDG PET/CT was 47.5 %, in which there were many false positive results as compared with those at visual diagnosis (Table 3 bottom). There were two false negative cases with ^18^F-FDG PET/CT; both were papillary adenocarcinomas with ground-glass opacity (*n* = 2, diameter: 25 and 25 mm, SUVmax: 1.89 and 2.00). The accuracy of ^11^C-MeAIB PET/CT with SUVmax = 2.0 cut-off value was 74.6 %. There were 13 false positive cases and 2 false negative cases. The false positive cases were as follows: granulomatous inflammatory mediastinal lymphadenopathy confirmed by surgical resection (*n* = 4), mediastinal sarcoid lymphadenopathy by lymph node biopsy (*n* = 3), IgG4-related syndrome (*n* = 1), and non-specific inflammatory change followed for more than 12 months (*n* = 5). The two false negative cases were papillary adenocarcinomas with ground-glass opacity (*n* = 2, diameter: 25 and 30 mm, SUVmax: 1.74 and 1.93). The former one was also false negative case with ^18^FDG, but the latter one was true positive with ^18^FDG.

### Lesion-based semi-quantitative diagnostic results

In the differential diagnosis of pulmonary nodules with SUVmax = 3.0 cut-off value, the accuracy of ^18^F-FDG PET/CT was 65.0 % (Table 4 top). The accuracy of ^18^F-FDG PET/CT for the diagnosis of mediastinal nodules (Table 4 bottom) was only 31.6 %. There were 26 cases of false positive results with ^18^F-FDG PET/CT, and the positive predictive value was only 27.8 %. ^11^C-MeAIB PET/CT showed better accuracy. The accuracy of ^11^C-MeAIB with SUVmax = 2.0 cut-off value for pulmonary nodules and mediastinal nodules was 76.2 and 76.3 %, respectively. In mediastinal nodules, there were only nine cases of false positive results, for a positive predictive value of 52.6 %.

**Table 4:** Lesion-based diagnostic results of FDG- and MeAIB-PET (PET/CT) using semi-quantitative analysis

Pulmonary nodules in the lung fields: *n* = 42									
FDG (SUVmax = 3.0 as threshold)					MeAIB (SUVmax-2.0 as threshold)				
	Malignant lesions, *n* = 22	Benign lesions, *n* = 20				Malignant lesions, *n* = 22	Benign lesions, *n* = 20		
FDG: positive	20	12	62.5	PPV (%)	MeAIB: positive	20	8	71.4	PPV (%)
FDG: negative	2	6	75.0	NPV (%)	MeAIB: negative	2	12	85.7	NPV (%)
	90.9	33.3	65.0			90.9	60.0	76.2	
	Sensitivity (%)	Specificity (%)	Accuracy (%)			Sensitivity (%)	Specificity (%)	Accuracy (%)	

Hilar and mediastinal nodules: *n* = 38									
FDG (SUVmax = 3.0 as threshold)					MeAIB (SUVmax-2.0 as threshold)				
	Malignant lesions, *n* = 10	Benign lesions, *n* = 28				Malignant lesions, *n* = 10	Benign lesions, *n* = 28		
FDG: positive	10	26	27.8	PPV (%)	MeAIB: positive	10	9	52.6	PPV (%)
FDG: negative	0	2	100.0	NPV (%)	MeAIB: negative	0	19	100.0	NPV (%)
	100.0	7.1	31.6			100.0	67.9	76.3	
	Sensitivity (%)	Specificity (%)	Accuracy (%)			Sensitivity (%)	Specificity (%)	Accuracy (%)	

* PPV positive predictive value, ** *NPV* negative predictive value

## Discussion

Several amino acid compounds have been suggested as feasible candidates for oncologic PET tracers which can overcome the drawbacks of ^18^F-FDG. ^11^C-MeAIB is considered to be one of the most promising amino acid radiotracers in clinical oncology. To our knowledge, the present study is the first to evaluate the clinical application of ^11^C-MeAIB PET-to-chest lesion diagnosis.

Our principal finding is that the diagnostic results of ^11^C-MeAIB PET/CT were better than those of ^18^F-FDG PET/CT, especially for the identification of non-malignant lesions. Table 3 clearly reveals the higher specificity of ^11^C-MeAIB PET/CT in these cases. In the evaluation of mediastinal lesions, ^11^C-MeAIB PET/CT showed 67.9 % specificity in lesion-based semi-quantitative diagnosis, while ^18^F-FDG PET/CT’s specificity was only 7.1 % (Table 4). The low positive predictive value (27.8 %) of ^18^F-FDG PET/CT confirms that positive uptake of ^18^F-FDG is not reliably diagnostic of malignancy. We believe that ^11^C-MeAIB PET/CT would make a great contribution in the diagnosis of patients with pulmonary nodules or mediastinal lesions, when CT and ^18^F-FDG PET/CT shows equivocal findings.

It should be noted that ^11^C-MeAIB PET/CT displayed high diagnostic accuracy in the evaluation of sarcoidosis. There were 20 sarcoid lesions in 14 patients and the average lesion ^18^F-FDG SUVmax was 7.3 ± 4.5, while that of ^11^C-MeAIB was 1.8 ± 0.7 (Table 5). Visual diagnosis with ^18^F-FDG PET/CT showed false positives in six patients, while those with ^11^C-MeAIB PET/CT showed false positives in three patients. Although ^18^F-FDG PET/CT can diagnose sarcoidosis by the specific uptake pattern of hilar and mediastinal lesions, sarcoidosis can form pulmonary nodules as well, and those lesions can be difficult to distinguish from malignancies. In addition, malignancy is often observed synchronously or metachronously in patients with sarcoidosis. In the present study, there were five pulmonary lesions (all of them finally confirmed as benign) in five patients with sarcoidosis. In these cases, ^11^C-MeAIB PET/CT played a useful diagnostic role (Fig. 3). Since ^11^C-MET accumulates in sarcoidosis [14, 15], it is suggested that ^11^C-MeAIB may be superior to ^11^C-MET in the differentiation of sarcoidosis from malignancy. Our result is compatible with previous studies using other amino acid PET tracers, such as [^18^F]-methyltyrosine (^18^F-FMT) [21]. Kaira et al. suggested in their report that the use of ^18^F-FMT PET in combination with ^18^F-FDG PET may be effective for this purpose. In terms of biological mechanism, it is not fully understood why ^11^C-MET and the other amino acid PET tracers (^11^C-MeAIB and ^18^F-FMT) show different uptake patterns in sarcoidosis. One of the conceivable mechanisms for the low uptake in sarcoidosis lesions of ^11^C-MeAIB and ^18^FMT is that these PET tracers, as artificial amino acids, are not metabolized in vivo [16, 22]. Concerning in vivo instability of ^11^C-MET, inflammatory lesion can be misdiagnosed by ^11^C-MET PET because of its non-specific accumulation of free ^11^C in blood when an inflammatory lesion shows hypervascularity. Comparative study of these amino acid PET tracers should be further evaluated.

**Table 5:** Patient characteristics (sarcoidosis)

Patient no.	Age/sex	Purpose of PET	Other diseases	ACE^b^ IU/L	Extra-pulmonary involvement of sarcoidosis	Treatment for sarcoidosis	Pathological Confirmation	Target lesion at PET	^18^F-FDG diagnosis			^11^C-MeAIB diagnosis		
									Visual diagnosis	SUVmax	Quantitative diagnosis^c^	Visual diagnosis	SUVmax	Quantitative diagnosis^d^
1	48/F	P&M	None	21.3	None	Not treated	Follow-up more than 1 year	Mediastinal lesions	TN	6.8	FP	TN	2.4	FP
								Pulmonary lesion	TN	1.3	TN	TN	0.6	TN
2	58/M	P&M	Ulcerative colitis	7.0	None	Not treated	Follow-up more than 1 year	Mediastinal lesions	TN	6.8	FP	TN	1.5	TN
								Pulmonary lesion	TN	0.8	TN	TN	0.7	TN
3	61/F	P&M	None	13.3	Eye	Steroid eye drop	Biopsy at thoracoscopy	Mediastinal lesions	FP	6.4	FP	TN	1.8	TN
								Pulmonary lesion	TN	1.3	TN	TN	0.7	TN
4	63/M (Fig. 3)	P&M	Diabetes (controlled)	31.9	Heart (diagnosed by PET)	Not treated	Biopsy at thoracoscopy	Mediastinal lesions	FP	7.6	FP	TN	1.7	TN
								Pulmonary lesion	FP	12.3	FP	TN	1.7	TN
5	36/M	P&M	None	13.4	Superficial lymph nodes	Not treated	Biopsy at superficial lymph node	Mediastinal lesions	FP	8.1	FP	FP	3.2	FP
								Pulmonary lesion	FP	10.9	FP	FP	2.1	FP
6	64/F	M	Gastric malignant lymphoma	19.1	None	Not treated	Follow-up more than 1 year	Mediastinal lesions	TN	4.1	FP	TN	1.8	TN
	65/F^a^	M	Gastric malignant lymphoma	23.9	None	Not treated	Follow-up more than 1 year	Mediastinal lesions	TN	6.1	FP	TN	2.0	TN
7	34/F	M	None	19.6	Superficial lymph nodes	Not treated	Biopsy at superficial lymph node	Mediastinal lesions	FP	17.9	FP	FP	3.6	FP
8	71/M	M	Colon polyps	24.6	None	Not treated	Follow-up more than 1 year	Mediastinal lesions	TN	5.7	FP	TN	1.9	TN
9	50/M	M	None	16.9	None	Not treated	Biopsy at thoracoscopy	Mediastinal lesions	TN	16.1	FP	TN	1.7	TN
10	70/F	M	Angina pectoris	11.1	None	Not treated	Follow-up more than 1 year	Mediastinal lesions	TN	2.8	TN	TN	1.7	TN
11	64/F	M	None	13.4	None	Not treated	Follow-up more than 1 year	Mediastinal lesions	TN	9.7	FP	TN	1.8	TN
12	77/F	M	None	23.7	Eye	Steroid eye drop	Biopsy at thoracoscopy	Mediastinal lesions	FP	7.3	FP	FP	2.0	FP
13	71/F	M	None	16.9	None	Not treated	Follow-up more than 1 year	Mediastinal lesions	TN	8.0	FP	TN	1.9	TN
14	44/F (Fig. 2)	M	Gastric malignant lymphoma	12.0	None	Not treated	Biopsy at thoracoscopy	Mediastinal lesions	FP	6.0	FP	TN	1.8	TN
								Average ± SD	7.3 ± 4.5			1.8 ± 0.7		
								Lesion-based diagnosis	TN: 12		TN: 4	TN: 16		TN: 15
									FP: 8		FP: 16	FP: 4		FP: 5
								Specificity:	60 %		8 %	80 %		75 %
								Patient-based diagnosis	TN: 9		TN: 1	TN: 12		TN: 11
									FP: 6		FP: 14	FP: 3		FP: 4
								Specificity:	60 %		7 %	80 %		73 %

*FP* false positive, *TN* true negative

^a^Same patient diagnosed 1 year later (with post-treated lymphoma)

^b^ACE: angiotensin-converting enzyme (normal range: 8.3 to 21.4 IU/L)

^c^Threshold: SUVmax = 3.0

^d^Threshold: SUVmax = 2.0

**Fig. 6:**
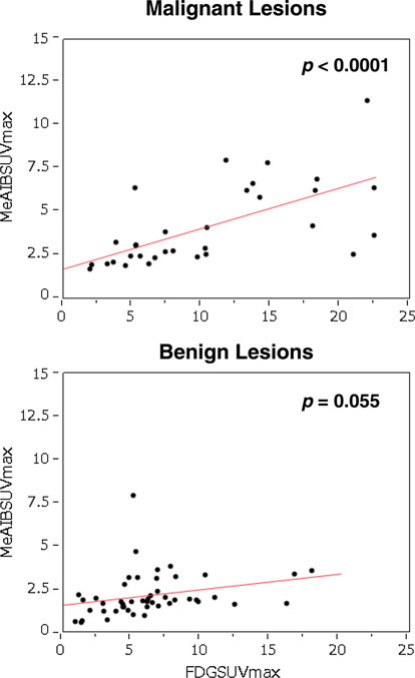
Relationship between SUVmax of ^11^C-MeAIB and that of ^18^F-FDG of each lesion in both PET study using Logistic regression. In malignant lesions, SUVmax of ^11^C-MeAIB shows a significant linear relationship with that of ^18^F-FDG (*p* < 0.0001, *R* ^2^ = 0.406). On the other hand, that of ^11^C-MeAIB also shows a linear correlation with that of ^18^F-FDG but not significant in benign lesions (*p* = 0.055, *R* ^2^ = 0.078)

In the diagnosis of malignancy, the sensitivity of ^18^F-FDG and ^11^C-MeAIB based on semi-quantitative patient-based diagnosis showed the same values (90.9 %) (Table 4). In addition, the uptake of ^11^C-MeAIB correlated well with ^18^F-FDG uptake and there were basically no discrepant cases (Fig. 6). ^18^F-FDG SUVs in malignant cases was usually two to three times higher than those of ^11^C. In previous studies using ^11^C-MET and ^18^F-FMT, ^11^C-MET and ^18^F-FMT SUVs were also two to three times lower than those of ^18^FDG [21, 23–25]. This may be a common drawback of amino acid PET tracers. Although our group included several different types of lung cancers, such as adenocarcinoma, squamous cell carcinoma, and small cell carcinoma, there was no significant difference in the uptake intensity of ^11^C-MeAIB among the different histological types. It is not what we anticipated for ^11^C-MeAIB PET/CT’s use as a predictor of therapeutic effect, because amino acid transporters are known to work as carriers of chemotherapeutic agents, such as cisplatin, methotrexate, taxol, and melphalan [26–28]. The role of ^11^C-MeAIB PET/CT as an imaging modality for patient-tailored medicine is unknown. Further study of pre- and post-chemotherapeutic ^11^C-MeAIB PET or PET/CT is needed.

Another drawback of ^11^C-MeAIB is its high physiological uptake by liver. It means that ^11^C-MeAIB PET/CT cannot be performed as a first-choice diagnostic modality in the evaluation of chest malignancies, because liver metastasis is common in lung cancer. Therefore, ^11^C-MeAIB PET or PET/CT cannot be performed as a study for staging of advanced lung cancer. This is why we focused our study only on the differential diagnosis in chest diseases, and excluded cases with apparent distant metastasis and direct invasion of neighboring organs.

## Conclusions

^11^C-MeAIB PET/CT was useful in the differential diagnosis of pulmonary and mediastinal mass lesions found on CT. ^11^C-MeAIB PET or PET/CT showed higher specificity than that of ^18^F-FDG PET/CT in differentiating between benign and malignant disease. Our data suggest that the combination of ^18^F-FDG and ^11^C-MeAIB may improve the evaluation of chest lesions, when CT and ^18^F-FDG PET/CT are equivocal.
